# Cocoon Syndrome: A Rare Cause of Bowel Obstruction Revealing Hidden Intestinal Tuberculosis

**DOI:** 10.7759/cureus.77090

**Published:** 2025-01-07

**Authors:** Anass Barchid, Walid Fadil, Anas Ahallat, Younes Aggouri, Said Aitlaalim

**Affiliations:** 1 General Surgery, Tangier University Hospital, Abdelmale Essaadi University, Faculty of Medicine, Tangier, MAR; 2 General Surgery, University Hospital Center, Tangier, MAR; 3 General Surgery, University Hospital Mohammed VI, Tangier, MAR

**Keywords:** bowel obstruction, cocoon syndrome, general surgery, intestinal tuberculosis, tuberculosis

## Abstract

Abdominal cocoon syndrome, also known as sclerosing encapsulating peritonitis (SEP), is characterized by the entrapment of intestinal loops by a fibrous membrane and is a rare cause of small bowel obstruction. We report a rare case of SEP secondary to intestinal tuberculosis in a 35-year-old male patient with no relevant medical history. He presented with symptoms suggestive of bowel obstruction, including acute abdominal pain, vomiting, and abdominal distension. Imaging showed pneumoperitoneum and encapsulation of the small intestine, while surgery revealed a fibrous capsule surrounding the bowel. Histopathological examination confirmed intestinal tuberculosis with granulomatous inflammation and caseating necrosis. Treatment involved surgical resection, ileostomy, and antitubercular therapy. This case emphasizes the importance of considering abdominal tuberculosis in the differential diagnosis of SEP.

## Introduction

Abdominal cocoon syndrome is characterized by the entrapment of intestinal loops by a fibrous membrane, giving the appearance of a sac-like structure [1]. Multiple etiologies have been linked to secondary SEP, but it remains a rare presentation of abdominal tuberculosis [1]. Intestinal tuberculosis may involve the intestine, peritoneum, or other visceral organs, representing a rare form of extrapulmonary tuberculosis [2]. Tuberculosis remains a significant health concern in many countries, particularly in tropical regions [3]. Intestinal tuberculosis manifestations are variable, ranging from ascites to peritoneal adhesions and fibrosis [4]. However, it may rarely be asymptomatic. In rare cases, the fibrosis can become extensive, forming a membranous structure that covers intestinal loops and causes bowel obstruction [1]. The diagnosis is primarily established using clinical, biological, and histomorphological findings. When tuberculosis is the etiology, there is no evidence to suggest that antitubercular therapy aids in the treatment of fibrous membranes. Medical treatment alone may fail, and surgery may be required [5].

We present a rare and unusual case of intestinal tuberculosis manifesting as sclerosing encapsulating peritonitis in a young male patient.

## Case presentation

We present the case of a 35-year-old male patient with no relevant medical history, who presented to the emergency department with sudden onset of diffuse abdominal pain, two episodes of vomiting, absence of stool and gas passage, and general malaise. On examination, there was generalized abdominal tenderness and palpable subcutaneous emphysema. Laboratory results revealed a slight decrease in hemoglobin (11.8 g/dL) with a normal platelet count (135,000/mm³) and white blood cell count (6,000/mm³). C-reactive protein (CRP) was elevated at 273 mg/L, indicating inflammation. Electrolytes and renal function were within normal limits, with urea and creatinine levels measured at 0.36 g/L and 10.2 mg/L, respectively.

**Figure 1:**
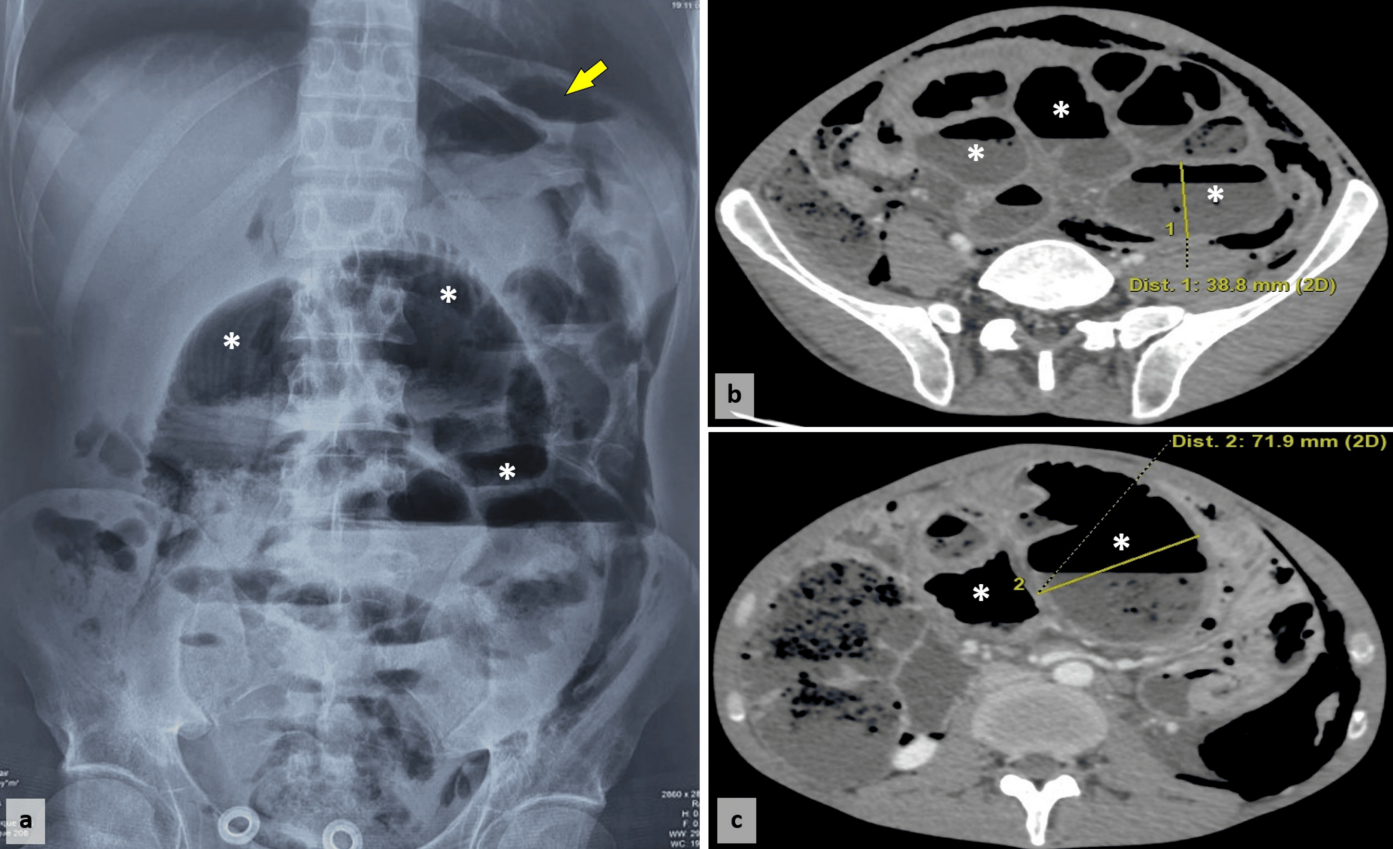
Abdominal X-ray showing marked gaseous distension of bowel loops (*), suggestive of bowel obstruction, along with pneumoperitoneum (arrows) (a). Axial abdominal CT scan showing multiple air-fluid levels and distended intestinal loops (*) (b and c).

X-ray revealed pneumoperitoneum and multiple air-fluid levels (Figure 1a). A CT scan showed a large pneumoperitoneum, a wall defect in an intestinal loop in the left lower quadrant, mesenteric fat infiltration, and intra-abdominal fluid. Small bowel distension was noted, with loops appearing encapsulated within a thin, contrast-enhanced peritoneal capsule, resembling a cocoon (Figure 1b, 1c). Additional findings included necrotic mesenteric and lumbar-aortic lymph nodes (the largest measuring 14 mm in the short axis). Subcutaneous emphysema was observed along the left abdominal wall, extending into the inguinal and left scrotal regions.

**Figure 2:**
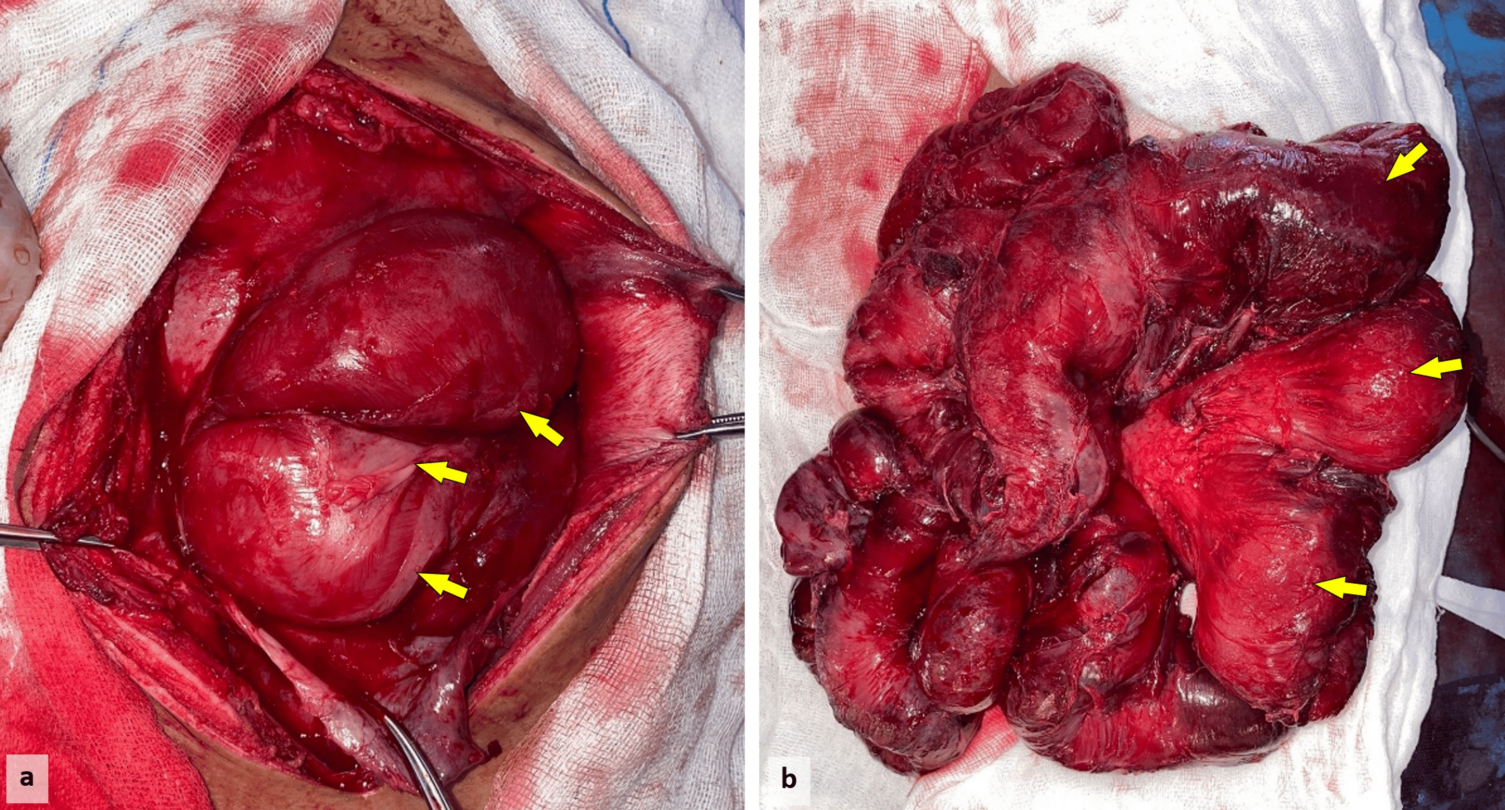
Intraoperative images showing a fibrous membrane covering the intestinal loops (arrows) (a). The resected specimen demonstrates significant congestion and hemorrhagic discoloration. The bowel loops appear distended (arrows) (b).

During surgery, there was an adhesive abdomen with a fibrous capsule surrounding the entire small intestine, with multiple adhesions between the loops of the small intestine and between the intestine and the capsule (Figure 2).

**Figure 3:**
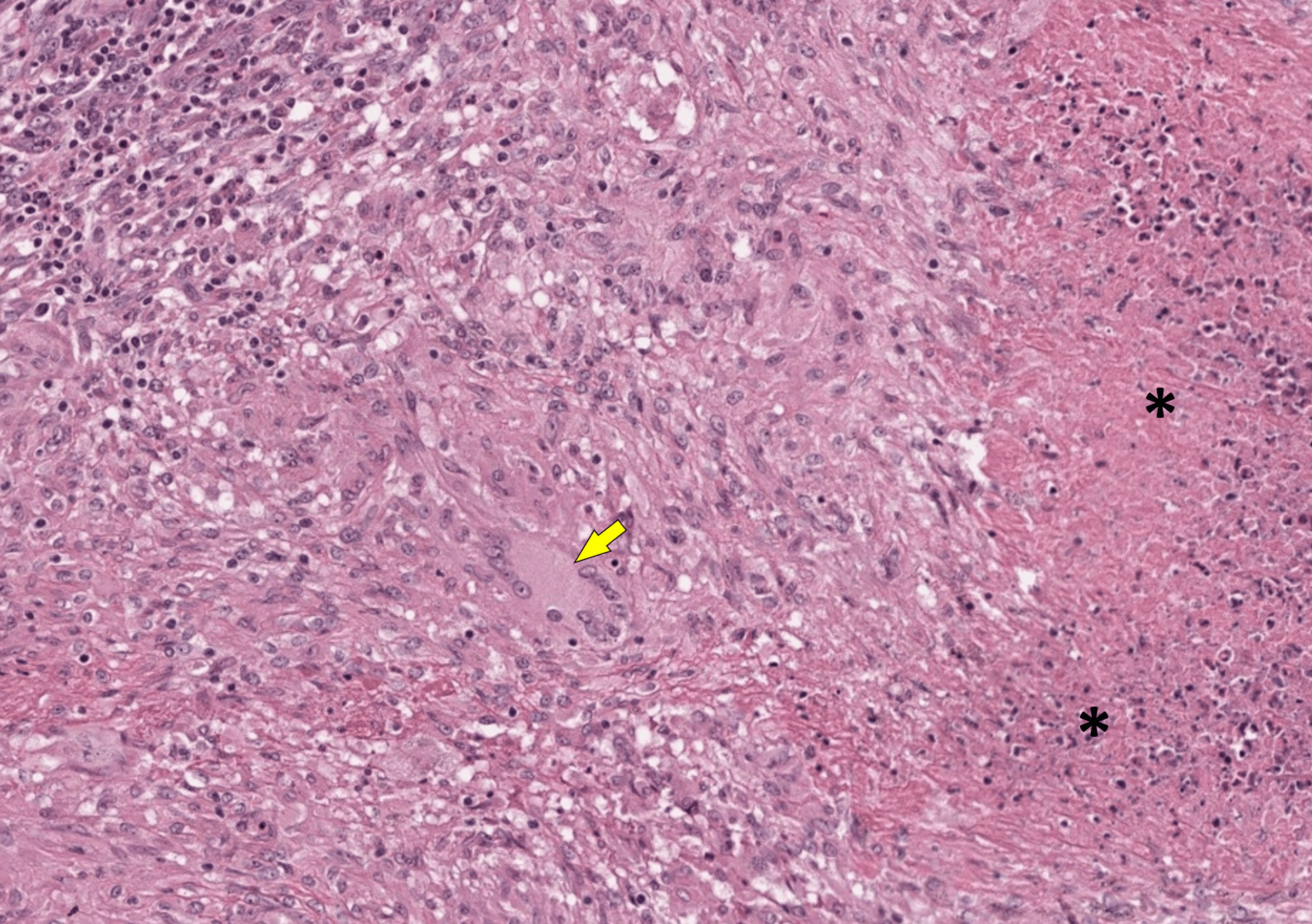
Photomicrograph showing granulomatous inflammation with multinucleated giant cells (arrow) and epithelioid macrophages, along with scattered lymphocytes. Areas of caseous necrosis are seen on the right (*) (H&E, × 200). H&E: hematoxylin and eosin

No purulent effusion or false membranes were observed. Intestinal distress was noted over 80 cm, starting 30 cm from the first jejunal loop, with a perforation at 50 cm from the first jejunal loop, causing leakage of digestive fluid. The entire small intestine was examined, and a resection of the distressed loop was performed, followed by the creation of a double ileostomy (one on the right and one on the left) due to intestinal retraction. Lavage with saline solution and drainage were also carried out, along with antitubercular therapy postoperatively. Histomorphological examination of the resected specimen revealed intestinal tuberculosis with a granulomatous reaction and characteristic caseating necrosis (Figure 3).

Overall, the postoperative recovery was uneventful, with no complications, and the patient was prepared for discharge once sufficient nutrition and pain management were established.

## Discussion

Abdominal cocoon or SEP is an uncommon cause of intestinal obstruction and a rare presentation of intestinal tuberculosis. It is characterized by the encasement of the small bowel by a thick fibrous membrane and may rarely affect other abdominal organs such as the large intestine, stomach, and liver [6]. SEP can be either idiopathic, with no apparent cause, or secondary, caused by a variety of etiologies including peritoneal dialysis, malignancy, long-term beta-blocker use, organ transplantation, and cirrhosis [1,6]. Tuberculosis is rarely associated with SEP. Evidence of associated pulmonary tuberculosis may aid in orienting the diagnosis, but it is not always present [5].

Depending on the rate of progression, clinical manifestations range from chronic abdominal pain, nausea, anorexia, vomiting, constipation, and weight loss to more serious presentations involving bowel obstruction and peritonitis due to perforation [6]. Sometimes, an abdominal mass may be present [7]. In our case, the patient had reported previous episodes of nausea and mild abdominal pain, which spontaneously resolved before the onset of peritonitis. Laboratory findings can aid in identifying a potential etiology. For tuberculosis, GeneXpert assay is highly sensitive and can be performed using specimens such as sputum or bronchoalveolar lavage if pulmonary tuberculosis is present. Interferon-gamma (IFN-γ) release assays have also been shown to be highly accurate [8].

Imaging techniques reveal dilated small intestinal loops with multiple air-fluid levels on abdominal X-rays [6]. A CT scan is a valuable diagnostic tool, showing a midline clustering of small intestine segments encased by a dense capsule with a contrast-free border, which is highly suggestive of SEP [9]. It may also show ascites, mesenteric infiltration, and peritoneal calcification [6]. However, the diagnosis of cocoon syndrome is most often made during surgery when the encasement of the small bowel is visualized [10]. Some findings may suggest a tubercular origin, such as caseating mesenteric lymph nodes and peritoneal tubercles [10]. However, a definitive diagnosis of tuberculosis is achieved through histopathological examination. Differential diagnoses mainly include other causes of intestinal obstruction, such as intussusception and intestinal malrotation [6], which are easily ruled out using imaging techniques.

Treatment involves effective antitubercular medication and management of intestinal obstruction, including nasogastric aspiration and intravenous fluids. In patients who fail medical therapy, surgical intervention, including resection of the fibrous membrane and adhesiolysis, may be necessary. In our case, the patient presented with a complicated bowel obstruction, requiring urgent resection of the obstructed intestine and continued antitubercular therapy.

## Conclusions

Small bowel obstruction caused by SEP is a rare event, and its association with intestinal tuberculosis is even more uncommon. SEP is characterized by a fibrous membrane encasing the small intestine. CT imaging is crucial for diagnosing this condition. This case report underscores the importance of considering abdominal tuberculosis in the differential diagnosis of acute abdominal conditions.
